# Apport de l'orientation clinique dans le diagnostic des hypertensions arterielles endocriniennes

**DOI:** 10.11604/pamj.2014.18.171.1619

**Published:** 2014-06-20

**Authors:** Siham El Aziz, Asma Chadli, Fatima Louda, Hassan El Ghomari, Ahmed Farouqi

**Affiliations:** 1 Unité d'Endocrinologie (p26), Centre hospitalo-universitaire Ibn Rochd, Casablanca, Maroc

**Keywords:** hypertensions arterielles endocriniennes, Syndrome de cushing, phéochromocytome, hyperaldostéronisme primaire, Endocrine arterial hypertension, Cushing syndrome, pheochromocytoma, primary aldosteronism

## Abstract

L'hypertension artérielle d'origine endocrinienne représente une cause curable d'hypertension artérielle (HTA), ce qui en justifie le dépistage. Une orientation clinique est souhaitable afin de prescrire les bilans adéquats, notamment dans les pays en voie de développement. L'objectif de notre étude est décrire les aspects cliniques des patients ayant une HTA endocrinienne diagnostiquée au CHU de Casablanca. Méthodes: Il s'agit d'une étude descriptive rétrospective de 2004 à 2011, incluant tout patient ayant une HTA endocrinienne (en dehors des dysthyroïdies) admis en Endocrinologie. Les données concernant les facteurs cliniques, paracliniques et thérapeutiques ont été colligées. Au cours de la période étudiée, 53 cas d'HTA endocrinienne ont été admis, avec une age moyen au diagnostic de 39,3 ans et un sex-ratio F/H de 3,9. Trente patients (56.6%) ont un age ont un âge inférieur à 40 ans. On ne retrouve pas d'hérédité tensionelle chez 88.7% des patients. Les étiologies sont le Syndrome de cushing (41.5%), le phéochromocytome (32%), les adénomes somatotropes (15%) et les hyperaldostéronismes primaire (7.5%). L'HTA est essentiellement de grade un ou deux (69.8%), avec un retentissement cardio-vasculaire chez 11 patients (20.7%). Les patients ayant un SC avaient tous des signes cutanés spécifiques. Il s'agissait de sujets jeunes avec plusieurs facteurs de risque cardiovasculaire. Nous avons retrouvé un amaigrissement chez 37.5% des patients ayant un phéochromocytome avec un indice de masse corporel moyen de 20 kg/m^2^. L'HTA était permanente dans 5 cas de phéochromocytomes. Il n'existait pas d'hypokaliémie chez 50% des patients ayant un hyperaldostéronisme. Les signes cliniques des patients ayant une acromégalie étaient francs, avec un age moyen plus élevé de 50 ans. Nous avons noté une régression de l'HTA chez 59% de ces patients après guérison de l'endocrinopathie en cause. Plusieurs signes cliniques peuvent nous orienter vers une HTA endocrinienne, notamment chez l'adulte jeune, et devraient indiquer les bilans adéquats afin d’éviter une méconnaissance du diagnostic.

## Introduction

L'hypertension artérielle est une pathologie fréquente atteignant près d'un quart de la population mondiale [1] avec près de 18% de la population atteinte au Maroc [2]. La gravité de L'HTA est due à la morbi-mortalité cardiovasculaire [3, 4] et aux complications occulaires et rénales[5]. Les hypertensions artérielles endocrines constituent des causes rares d'HTA mais ce sont des causes facilement décelables et curables [6–8]. De plus, il s'agit le plus souvent d'HTA résistantes touchant le sujet jeune. Les HTA endocrines sont représentées par l'hyperaldostéronisme primaire, les dysthyroïdies, le syndrome de Cushing, le phéochromocytome, l'acromégalie, les blocs enzymatiques ou encore l'hyperparathyroïdie primaire [9]. L'importance de diagnostiquer une HTA endocrinienne réside dans le fait que l'HTA, pathologie chronique et incurable peut devenir après un éventuel traitement étiologique curable, d'où une diminution de la morbi-mortalité avec évidemment une qualité de vie nettement améliorée et des couts de santé moindres. De plus, même en l'absence de traitement étiologique possible, la connaissance du mécanisme physiopathologique de cette HTA permet de prescrire des traitements spécifiques et adaptés [10].

Nous avons pris en charge 80 cas d'endocrinopathies, hors diabète et dysthyroïdies, durant la période janvier 2001 à Mars 2011, parmi lesquelles nous retrouvons 53 cas d'HTA endocriniennes. L'objectif de notre étude est de décrire les manifestations cliniques des patients ayant une HTA endocrinienne suivis au Centre hospitalo-universitaire de Casablanca de 2004 à 2011, ce qui constitue une des plus grandes expériences dans notre pays. Certaines conclusions cliniques pourraient aider au diagnostic de l'HTA endocrinienne, ce qui est sans aucun doute utile au Maroc vu l'accessibilité et le cout des dosages hormonaux chez une population en majorité ne disposant pas de couverture sociale.

## Méthodes

**Type d’étude:** Il s'agit d'une étude descriptive rétrospective.

### Population étudiée

Entre Janvier 2004 et Mars 2011, une HTA endocrinienne a été identifiée chez 53 patients référés à notre unité. Ont été inclus les patients ayant une hypertention artérielle retenue sur les critères du Joint National Comittee 2003 [11], et ayant une des endocrinopathies suivantes: Syndrome de Cushing endogène, phéochromocytome, hyperaldostéronsime primaire, acromégalie et hyperparathyroïdie. Nous avons exclu les patients ayant une HTA associée à une dysthyroïdie ainsi que les patients ayant un diabète de type 2 ou de type 1 associé à une HTA sans autre endocrinopathie causale. Les patients ayant une hyperplasie congénitale des surrénales sont également exclus vu le suivi en pédiatrie.

### Définition des variables

L'HTA a été définie selon les recommandations du Joint National Comittee 2003 JNC-VII [11] comme une pression artérielle systolique ‘ 140 mmHg et /ou une pression artérielle diastolique ‘ 90 mmHg. L'HTA est considérée comme paroxystique s'il existe des pics tensionnels documentés associés à des triades de céphalées-sueurs et palpitations.

L'HTA a été classée selon les recommandations de la société européenne d'hypertension artérielle en [12]:

- HTA grade 1 (HTA légère): Pression artérielle systolique (PAS) entre 140 et 159 mmHg et/ou pression artérielle diastolique (PAD) comprise entre 90 et 99 mmHg
- HTA grade 2 (HTA modérée): PAS entre 160-179 mmHg et/ou PAD entre 100-109 mmHg
- HTA grade 3 (HTA sévère): PAS ≥ 180 mmHg et/ou PAD’ 110 mmHg
- HTA systolique isolée: PAS ≥ 140 et PAD < 90 mmHg

Le diagnostic des endocrinopathies a été fait selon les habitudes usuelles, le diagnostic positif comportant:

Pour le phéochromocytome, un taux élevé de dérivés méthoxylés urinaires (par chromatographie). Le diagnostic de localisation comportait un examen scannographique surrénalien avec dans certains cas une scintigraphie à la MIBG, lors de la recherche de formes ectopiques.

Au cours du syndrome de Cushing, un taux élevé de cortisol libre urinaire sur les urines de 24 h sous réserve d'une créatinine urinaire normale.

Au cours de l'hyperaldostéronisme primaire, un taux élevé d'aldostérone avec activité rénine plasmatique basse.

Au cours de l'acromégalie, hormis la forte suspicion clinique, le diagnostic a été confirmé par l'existence d'un taux élevé d'IGF1 (insuline likegrowth factor 1); Le bilan morphologique reposait sur l'IRM hypothalamo-hypophysaire à la recherche d'un adénome hypophysaire.

Au cours de l'hyperparathyroïdie, un taux élevé de parathormone associé à une hypercalcémie et hypercalciurie.

### Collecte et analyse des données

La collecte des données s'est fait moyennant une fiche d'exploitation comportant les éléments suivants: données démographiques, caractéristiques de l'HTA: ancienneté, type, traitement, retentissement, données concernant l'endocrinopathie causale: signes cliniques, bilan hormonal, traitement et évolution. La saisie et l'analyse des données s'est fait grâce au logiciel sphinx-plus.

## Résultats

**Figure 1:**
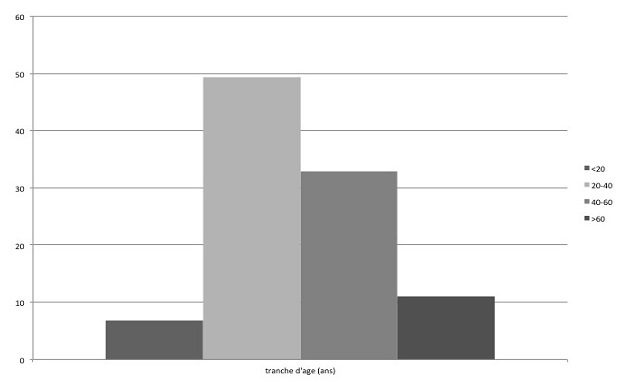
Age à la découverte du diabète

Cinquante trois patients ayant une HTA endocrinienne ont été inclus dans cette étude (40 femmes, 13 hommes, âge18-70 ans). Concernant les différentes étiologies retrouvées, nous notons 22 cas de syndrome de Cushing (41.5%), 17 phéochromocytomes (32%), 8 acromégalies (15%), 4 hyperaldostéronismes (7.5%) et deux cas d'hyperparathyroïdie primaire (3.4%). Les causes endocriniennes de ces hypertensions artérielles sont rapportées dans le Tableau 1. L’âge moyen au diagnostic de l'HTA d'origine endocrinienne était de 39.3ans, avec 56.7% des patients qui ont moins de 39 ans. La répartition des patients selon la tranche d’âge au diagnostic de l'HTA endocrinienne est rapportée dans la Figure 1. L'ancienneté présumée de l'HTA endocrinienne (considérée à partir du diagnostic de l'HTA) variait entre 6 et 144 mois avec une ancienneté moyenne de 24 mois. Le profil clinique des patients selon la sévérité de l'HTA est décrit dans le Tableau 2. Il n'existe pas d'hérédité tensionelle chez 88.7% de ces patients. L'existence d'autres facteurs de risque, hormis l'HTA, est retrouvée chez28 patients (52.3%). Le Tableau 3 illustre les caractéristiques des patients selon l’étiologie de l'HTA endocrinienne.

**Tableau 1:** Etiologies de l'HTA endocrinienne

	Nombre de cas	%
**Phéochromocytome**	17	32.1
**Syndrome de cushing**	22	41.5
**Hyperaldostéronisme**	4	7.5
**Acromégalie**	8	15.1
**Hyperparathyroidie**	2	3.8
**Total**	53	100

**Tableau 2:** Profil des patients selon la sévérité de l'HTA

**n**	12	25	6	3
**Femmes/Hommes**	9/3	19/6	5/1	3/0
**TAS (mmhg)**	145	175	185	167
**Nombre moyen de traitement anti-hypertenseurs**	1.5	1.4	3	2
**Age à la découverte de l'HTA (ans)**	35	40	38	42
**Age (ans)**	37	42	41	47

**Tableau 3:** Données cliniques et paracliniques selon l’étiologie en cause de l'HTA endocrinienne

	Phéochromocytome	Hyperaldostéronisme	Syndrome de cushing	Acromégalie	hyperparathyroidie
**Nombre de cas**	17	4	22	8	2
Sexe (%)					
F	70,6	100	68.2	87,5	100
M	28,3	0	31.8	12,5	0
Age moyen (ans)	41.12	40	36.7	50	44,5
Ancienneté HTA (ans)	2.64	5	2	1	1
IMC moyen (kg/m^2^)	20.3	28	29	28.3	32,1
Hypokaliémie (%)	0	75	22.7	0	0
Dyslipidémie (%)	14.3	75	36.4	25	50
Obésité (%)	0	25	36.4	37,5	100
Diabète (%)	11.8	0	54.5	37,5	0

**Figure 2:**
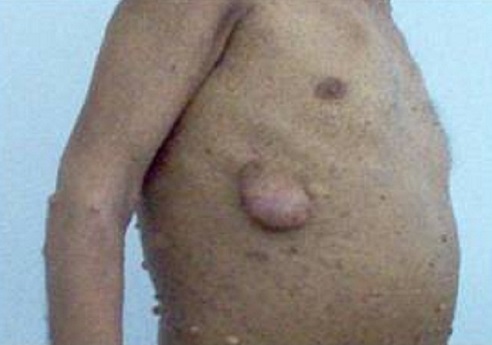
Lentigines et neurofibromes cutanés

Le mode de découverte du phéochromocytome était l'HTA chez 16 patients (94.2%), et dans un cas il s'agissait d'un bilan de néoplasie endocrinienne multiple révélant un phéochromocytome. Un amaigrissement était retrouvé chez six patients (37.5%) avec un indice de masse corporel moyen de 20 kg/m^2^. Le phéochromocytome était surrénalien dans 16 cas (94.1%), unilatéral chez 14 patients. On a retrouvé un phéochromocytome bilatéral chez 2 patients, et un phéochromocytome ectopique à localisation vésicale chez une patiente. Le type d'HTA au cours du phéochromocytome était retrouvé dans 10 dossiers avec 5 cas d'HTA permanente (50%) et 5 cas d'HTA paroxystique. Le bilan de néoplasie endocrinienne multiple réalisé chez 11 patients ayant un phéochromocytome retrouve trois cas de NEM2A et un cas de NEM 2B avec neurofibromes et lentigines à l'examen clinique (Figure 2). Concernant les quatre cas d'hyperaldostéronisme, il s'agissait de quatre patients de sexe féminin, tous âgés de moins de 50 ans, avec une HTA sévère chez deux d'entre eux. L'hypokaliémie est retrouvée dans trois cas et nous avions une patiente âgée de 35 ans ayant un taux de kaliémie normal, dont l'hyperaldostéronisme a été découvert devant une HTA avec incidentalome. L'hyperaldostéronisme était du chez ces quatre patientes à un adénome de Conn, traité chirurgicalement.

**Figure 3:**
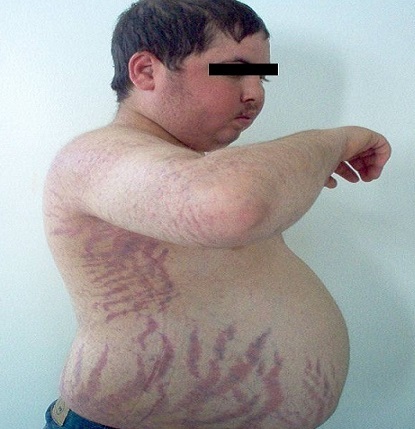
Signes cutanés du syndrome de cushing

Les patients ayant un syndrome de Cushing ont tous des manifestations cutanées franches (Figure 3) avec une obésité facio-tronculaire et érythrose faciale visibles dès l'inspection clinique. L’âge moyen de ces patients est de 36 ans avec 68% de femmes. Un diabète est retrouvé chez 54.5% de ces patients jeunes et une dyslipidémie chez 37% des patients. L'hypokaliémie est notée chez cinq patients (22.7%); L'ancienneté des signes cliniques cutanés est de 4 ans en moyenne et l'ancienneté de l'HTA est de 2 ans. Il s'agit donc la plupart du temps de sujets jeunes avec des signes cutanés marqués, avec plusieurs facteurs de risque cardiovasculaire associés à l'HTA.

**Figure 4:**
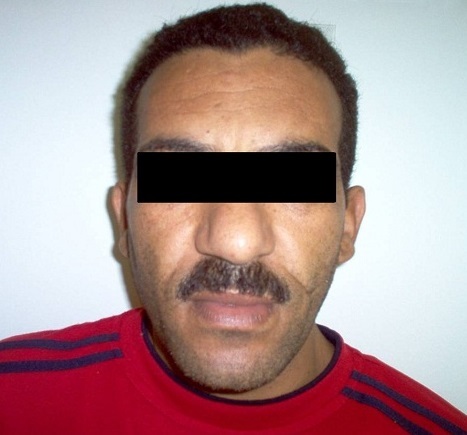
Aspect du visage chez un patient acromégale

**Figure 5:**
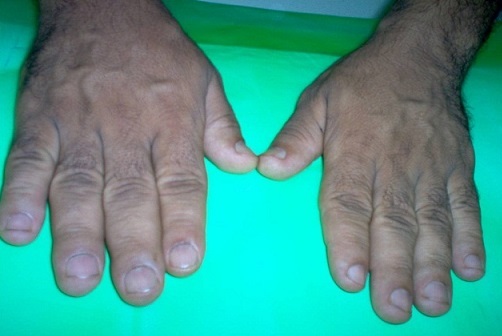
Modifications de l'aspect des mains chez un patient acromégalie

La prévalence de l'HTA au cours de l'acromégalie dans notre série est de 40%. Nous avons ainsi huit patients ayant une HTA grade 1 ou 2, sans hérédité tensionelle, ayant un âge moyen de 50 ans, et des signes cliniques très évocateurs d'acromégalie (Figure 4, Figure 5).

Les deux patients ayant une hyperparathyroïdie sont des femmes avec un âge moyen de 44 ans, ayant une HTA diagnostiquée depuis 1 an, et dont le mode de découverte de l'hyperparathyroïdie était les fractures osseuses.

Le retentissement cardiovasculaire de l'HTA était une cardiomyopathie hypertensive chez neuf patients (45%) et une cardiopathie ischémique chez deux patients (10%). Des signes de rétinopathie hypertensive ou diabétique sont retrouvés chez 58.3% des patients.

Concernant le suivi des patients, nous avons noté un suivi correct après traitement étiologique chez 22 patients, avec régression de l'HTA après guérison de l'endocrinopathie causale chez 13 patients (59%).

## Discussion

L’ HTA endocrinienne devrait être recherchée en cas d'HTA du sujet jeune, d'HTA résistante ou d'HTA avec hypokaliémie. Les symptômes cliniques sont très divers et une bonne connaissance des symptômes les plus évocateurs est souhaitable afin d'orienter les investigations paracliniques adaptées [9]. Ainsi, l'interrogatoire et l'examen clinique devraient être minutieux avant l'instauration d'un traitement anti-hypertenseur [13]. En effet, ces HTA endocrines ont un plus mauvais pronostic cardiovasculaire en raison des complications plus fréquentes [14]. De plus, il existe une possibilité de traitement spécifique ou voire même de guérison de l'HTA, après traitement étiologique [15].

Vu que notre étude est rétrospective, nous pouvons noter certaines limites notamment les données manquantes sur l'examen clinique et les éléments de suivi des patients.

Dans notre série, l'endocrinopathie en cause la plus retrouvée est le Syndrome de Cushing ainsi que le phéochromocytome avec une prévalence respective de 41.5% et 32%. Nous n'avons que 4 cas d'HAP, alors que selon la littérature l'HAP est présent chez près de 10% des patients hypertendus [16–19]. Il s'agit le plus souvent d'hypertension artérielle résistante [16, 20] avec hypokaliémie présente chez 20 à 50% des patients [21, 22]. Ainsi le diagnostic d'HTA endocriniennes du à une HAP devrait être évoqué devant une HTA résistante, une HTA avec hypokaliémie, une HTA associée à un nodule surrénalien et en cas d'HTA chez le sujet jeune [23–25]. Dans notre série, nous avons évoqué le diagnostic d'HAP devant une HTA associée à une hypokaliémie chez trois patients sur quatre, de plus il s'agit de sujets relativement jeunes avec un âge moyen de 40ans et un âge moyen à la découverte de l'HTA de 35ans. Nous avons un cas d'HTA sans hypokaliémie avec existence d'un incidentalome. Il existe probablement un sous diagnostic des cas d’ hyperaldostéronisme vu l'absence de dosage systématique du bilan hormonal en cas d'HTA correspondant aux critères de dépistage.

Le phéochromocytome est souvent cliniquement très évident vu la triade céphalées/sueurs/palpations et les accès paroxystiques d'HTA souvent décrits [26]. Cependant, il peut s'agir d'une HTA permanente dans près de 50% des cas [26], ce qui dans notre série a été retrouvé chez 5 patients (50%). Dans la série allemande concernant 201 phéochromocytomes [27, 28], 32.4% des patients n'avaient pas la triade paroxystique. Un des signes d'appel clinique évocateurs, hormis la classique triade de Menard est l'amaigrissement. Contrairement à l'HTA essentielle, survenant le plus souvent chez un sujet en surpoids ou obèse, nous retrouvons un IMC moyen de 20.3 kg/m^2^ avec un amaigrissement retrouvé chez 37.5% des patients. Dans la série multicentrique allemande [29], un amaigrissement est noté chez 17.3% des patients. Le phéochromocytome est le plus souvent retrouvé chez un sujet jeune avec un âge inférieur à 35 ans selon la série de 165cas de l'hôpital européen Georges Pompidou [30]. L’âge moyen est de 42 ans dans la série de Walters et al [31]. Concernant la prépondérance du sexe féminin retrouvée dans notre série, elle ne semble pas exister dans les autres séries beaucoup plus significatives statistiquement.

Une maladie familiale type néoplasie endocrinienne multiple est retrouvée chez près de 30% des patients [32]. Nous avons 27% de néoplasie endocrinienne multiple parmi nos phéochromocytomes. Le Tableau 4 compare les signes cliniques retrouvés chez les patients ayant un phéochromocytome selon différentes séries.

**Tableau 4:** Signes cliniques orientant vers le phéochromocytome comparé avec des données de certaines séries

	Amarl. [33]	Koptche et al [30]	Marelli et al [29]
**Nombre de patients**	n= 192	n=201	n=284
**Sexe**			
F		(55.7)	53.6%
M	89 (46,4)		46.4%
**HTA**			
Oui	171 (89.1)	(93.9%)	
Non		(6.1)	21.1%
Permanente	-	5.9%	
Paroxystique	-	35.9%	59.7%
IMC moyen (kg/m^2^)	23.3 ± 4.4	--	-
Amaigrissement	--	17.9%	
NEM ou pathologie			
**Familiale**		--	--
Oui	34 (17.7)		
Non			
Age moyen (ans)	43.9 ± 14.5	4.7 ± 1.3	-
Ancienneté HTA (ans)	3	1.7	-

L'HTA est présente chez quasiment 80% des patients ayant un syndrome de Cushing [33], et sa présence serait corrélée à l'ancienneté de l'hypercortisolisme [34–37]. Il s'agit le plus souvent d'une HTA grade 2, survenant chez un sujet relativement jeune, souvent obèse associant plusieurs facteurs du risque cardiovasculaire [35]. Il peut s'agir parois d'HTA sévère [7, 20], avec existence d'une hypokaliémie sévère. Les signes cutanés les plus spécifiques [36, 37], ont été retrouvés chez tous nos patients. Ils devraient être recherchés chez tout patient hypertendu, jeune, obèse, avec nombreux facteurs de risque cardiovasculaire [35].

L'HTA est retrouvé chez près de 40% des patients ayant une acromégalie [38, 39], généralement de grade 2 ou 3 [40]. Il s'agit le plus souvent des sujets plus âgés [39] et un des signes majeurs d'orientation clinique est le faciès acromégaloïde typique, visible lors de l'inspection avec des mains larges, avec doigts boudinés et un visage caractéristique.

## Conclusion

Ainsi, selon notre expérience clinique, on réalise que les patients ont quasiment tous des signes cliniques orientateurs d'HTA endocrine. Les patients ayant un syndrome de cushing, sont le plus souvent jeunes, cumulent les facteurs de risque et ont des signes cliniques cutanés très évocateurs. Les patients ayant un phéochromocytome sont généralement maigres, jeunes avec HTA paroxystique ou permanente. Enfin l'acromégalie est facilement reconnue vu l'aspect clinique des patients. Par ailleurs, Il existe probablement au Maroc un sous- diagnostic des hyperaldostéronismes en médecine de santé publique. Ainsi, selon quelques éléments cliniques, le praticien peut être orienté vers une HTA endocrinienne; C'est dire l'importance de l'enquête étiologique de l'HTA lors du diagnostic de l'HTA avant l'instauration d'un traitement anti-hypertenseur à vie notamment chez des sujets jeunes.
